# Supplementary material for: A high-resolution mRNA expression time course of embryonic development in zebrafish
Source: eLife. 2017 Nov 16;6:e30860. doi: 10.7554/eLife.30860 (PMC5690287; doi:10.7554/eLife.30860)
Supplement: Supplementary file 6. [file elife-30860-supp6.zip › biolayout-clusters-files/Cluster124-genes.html]

Cluster124


# Cluster124: Genes

| Ensembl ID | Gene Name | Chr | Start | End | Biotype |
| --- | --- | --- | --- | --- | --- |
| ENSDARG00000087345 | CABZ01059415.2 | 1 | 55990642 | 55992780 | protein\_coding |
| ENSDARG00000002311 | fabp11b | 16 | 16240784 | 16244222 | protein\_coding |
| ENSDARG00000090268 | krtt1c19e | 19 | 5422878 | 5429446 | protein\_coding |
| ENSDARG00000053364 | pax10 | 3 | 32414785 | 32434169 | protein\_coding |
| ENSDARG00000088589 | ponzr3 | 7 | 22403905 | 22406054 | protein\_coding |
| ENSDARG00000087440 | ponzr4 | 7 | 22409205 | 22413135 | protein\_coding |
| ENSDARG00000075713 | shox2 | 15 | 2218531 | 2224850 | protein\_coding |
| ENSDARG00000091996 | si:ch211-117m20.5 | 5 | 30014379 | 30015748 | protein\_coding |
| ENSDARG00000056292 | vsx1 | 17 | 21043567 | 21046269 | protein\_coding |
| ENSDARG00000012126 | zgc:109965 | 21 | 12969717 | 12988254 | protein\_coding |
